# Reproductive isolation caused by azoospermia in sterile male hybrids of *Drosophila*

**DOI:** 10.1002/ece3.6329

**Published:** 2020-05-04

**Authors:** Hunter Davis, Nicholas Sosulski, Alberto Civetta

**Affiliations:** ^1^ Department of Biology University of Winnipeg Winnipeg MB Canada

**Keywords:** azoospermia, *Drosophila*, hybrid male sterility, speciation, sperm transfer

## Abstract

Recently diverged populations in the early stages of speciation offer an opportunity to understand mechanisms of isolation and their relative contributions. *Drosophila willistoni* is a tropical species with broad distribution from Argentina to the southern United States, including the Caribbean islands. A postzygotic barrier between northern populations (North America, Central America, and the northern Caribbean islands) and southern populations (South American and the southern Caribbean islands) has been recently documented and used to propose the existence of two different subspecies. Here, we identify premating isolation between populations regardless of their subspecies status. We find no evidence of postmating prezygotic isolation and proceeded to characterize hybrid male sterility between the subspecies. Sterile male hybrids transfer an ejaculate that is devoid of sperm but causes elongation and expansion of the female uterus. In sterile male hybrids, bulging of the seminal vesicle appears to impede the movement of the sperm toward the sperm pump, where sperm normally mixes with accessory gland products. Our results highlight a unique form of hybrid male sterility in *Drosophila* that is driven by a mechanical impediment to transfer sperm rather than by an abnormality of the sperm itself. Interestingly, this form of sterility is reminiscent of a form of infertility (azoospermia) that is caused by lack of sperm in the semen due to blockages that impede the sperm from reaching the ejaculate.

## Graphical abstract

Recently diverged population in the early stages of speciation offer an opportunity to understand mechanisms of isolation. Here, we test isolating barriers and show that sterile male hybrids in crosses of *D. willistoni* transfer an ejaculate devoid of sperm. We identify new mechanisms of sterility caused by mechanical impediment to transfer sperm rather than by an abnormality of the sperm itself.

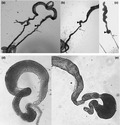

## 1 INTRODUCTION

Among sexually reproducing organisms, barriers that can impede interbreeding among individuals can contribute to reproductive isolation and speciation (Coyne & Orr, 2004; Mayr, 1942). While reproductive isolation mechanisms interact, barriers to gene flow can be broadly divided into those imposed by environmental conditions and considered extrinsic, or due to changes in the biology of individuals, independent of the external environment, and considered intrinsic (Bierne, Welch, Loire, Bonhomme, & David, 2011). Biological barriers that prevent hybridization can manifest themselves at premating stages (Jennings, Mazzi, Ritchie, & Hoikkala, 2011; Kozak, Reisland, & Boughmann, 2009; Nickel & Civetta, 2009; Svensson, Karlsson, Friberg, & Eroukhmanoff, 2007) or after mating has taken place. Postmating reproductive isolation can take place before fertilization through competitive or noncompetitive mechanisms (postmating prezygotic) (Gregory & Howard, 1994; Howard, Gregory, Chu, & Cain, 1998; Jennings, Snook, & Hoikkala, 2014; Price, 1997), or after fertilization due to reduced fitness of hybrid offspring (postzygotic) (Aalto, Koelewijn, & Savolainen, 2013; Haldane, 1922; Ishishita, Kinoshita, Nakano, & Matsuda, 2016; Liang & Sharakhov, 2019).

Different types of barriers can be critical to speciation. In *Drosophila*, studies on the rate at which different barriers evolve have shown that, on average, prezygotic isolation evolves faster than postzygotic isolation (Coyne & Orr, 1989, 1997), with premating barriers evolving faster than postmating prezygotic and postzygotic isolation being even slower (Turissini, McGirr, Patel, David, & Matute, 2018). However, the average rate of evolution of such barriers among species is not necessarily indicative that premating mechanisms are always more relevant in establishing isolation. For example, among Hawaiian species of *Drosophila*, the strength of premating versus postmating barriers can be dependent on sympatry versus allopatry status of the species (Carson, Kaneshiro, & Val, 1989; Kaneshiro, 1976; Kang, Garner, Price, & Michalak, 2017). Among populations of *Drosophila montana*, there is evidence that premating mechanisms contribute to isolation, but premating isolation increases with distance between populations, while postmating isolation is independent of distance, suggesting its important role in the early stages of speciation (Garlovski & Snook, 2018). While mechanisms of isolation have been studied extensively, they have been most commonly studied using species in which isolation is already fully established, thus making it difficult to differentiate between barriers that might evolve postspeciation from those that might have contributed to reduce gene flow in early stages of speciation. The identification of isolating barriers among diverging populations or partially isolated subspecies that have not yet reached a full‐species status can help address questions on the role of different isolating mechanisms in speciation. Moreover, it has become increasingly evident that proper identification of the speciation phenotype aids in understanding not only the speciation process but also its genetic basis (Mullen & Shaw, 2014). In turn, fine phenotypic characterization is crucial to functionally annotate genes.

*Drosophila willistoni* is a nonhuman commensal that uses flowers and fruits as substrates (Markow & O’Grady, 2008). The species was once believed to continuously spread from the southern United States to South America (*D. w. willistoni*), with a different subspecies (*D. w. quechua*) restricted to the west of the Andes in a narrow geographical area near Lima, Peru. It has been recently found that *D. w. willistoni* is subdivided into two partially isolated populations (subspecies) that are reproductively isolated from each other: *D. w. willistoni* in North America, Central America, and northern Caribbean islands, and *D. w. winge* in South America and southern Caribbean islands (Mardiros et al., 2016). When a female of *D. w. willistoni* mates with a male of *D. w. winge*, the resulting males are sterile, but the females are fertile. In the reciprocal cross, all offspring are fertile. It has also been previously determined that copulation duration is similar for sterile hybrid males and parental species and that the external male genitalia show no differences between the subspecies. Further, examination of the internal genitalia found no evidence of major atrophy in the hybrids relative to parental species, and the sterile males produced motile sperm but failed to place sperm within the female reproductive storage organs after mating (Civetta & Gaudreau, 2015). Whether hybrid male sterility due to failure to transfer sperm is unique to the two populations previously assayed (Civetta & Gaudreau, 2015) remains unclear. Moreover, we lack clear phenotypic characterization of what causes sterile male hybrids’ failure to transfer sperm and whether any form of assortative mating, or postmating prezygotic incompatibility, reduces gene flow between these two different subspecies.

Here, we use strains derived from different populations of the two subspecies (i.e., *D. w. willistoni*: Guadeloupe, Puerto Rico, and *D. w. winge*: Uruguay and Saint Vincent). We found assortative mating among individuals of the same populations and no evidence of noncompetitive postmating prezygotic isolation. Using a series of interrupted mating assays to track the fate of sperm and ejaculate of sterile male hybrids, we find that the sterile males manage to transfer an ejaculate that triggers the expected responses of elongation and expansion of the female uterus. However, the ejaculate is devoid of sperm. We identify a large mass forming a bulge at the basal end of the testes (*i.e*., the seminal vesicle) in sterile males that appears to impede the movement of the sperm toward the sperm pump, where sperm normally mixes with secretions produced by the accessory glands to produce the ejaculate. This mechanical impediment to transfer sperm represents a novel form of hybrid male sterility in *Drosophila*.

## 2 MATERIALS AND METHODS

### 2.1 Drosophila stocks and maintenance

Four strains obtained from the National *Drosophila* Species Stock Center were used in this study. The stocks were *Drosophila willistoni willistoni* 14030–0814.10 (wil(G): Guadeloupe) and 14030–0811.12 (wil(P): Toro Negro, Puerto Rico), and *D. w. winge* 14030–0811.13 (win(S): Saint Vincent and the Grenadines) and 14030–0811.16 (win(U): Rocha, Uruguay). A previous study established postzygotic isolation of Central America, North America, and northern Caribbean islands from South American and southern Caribbean island strains and suggested a subdivision of *D. w. willistoni* into two subspecies named *D. w. willistoni* and *D. w. winge* (Mardiros et al., 2016).

Throughout the experiments, flies were kept in either 8 oz. bottles containing 50 ml of cornmeal–yeast–agar–molasses (CYAM) medium or in 27 × 93mm (diameter × height) vials containing 6–8 ml of CYAM. Thirty females and thirty males were set up in bottles and kept in a 12:12‐hr light–dark cycle at 22–24°C. Emerging adult flies used in mating experiments, in interrupted mating assays or to produce hybrids, were collected under light CO_2_ anesthesia as newly emerged every 4 hr to ensure virginity. Virgin females and naïve males were separated, maintained at a density of 20 to 30 flies per vial, and aged for 5 to 6 days posteclosion before being used.

### 2.2 Premating isolation and fecundity

We measure premating isolation among strains of the same subspecies (i.e., wil(G) × wil(P); win(S) × win(U)) as well as different subspecies (i.e., wil(G) × win(U); wil(P) × win(S)) using multiple‐choice mating experiments (Jennings et al., 2011). Virgin females and naïve males from different strains were transferred without anesthesia from collection vials into vials with either red‐ or blue‐colored food and allowed to feed overnight. The dyes were alternated to account for possible dye effects. Flies were then placed together in bottles containing CYMA food supplemented with yeast in groups of 30 males and 30 females per strain for a total of 120 flies. Mating pairs were observed in the morning until half of all possible matings had occurred, but for no longer than an hour, mating pairs were removed and identified based on the color of their abdomen (Casares et al., 1998; Gilbert & Starmer, 1985). The experiments were run over 3–4 replicates (different days) of each cross. We analyzed whether females show preference in mating with males of the same population or of a different population by using a generalized linear mixed model (GLMM) with a binary response variable (package lme4, Bates, Maechler, Bolker, & Walker, 2015) using the R software 3.2.3 package (R Development Core Team, 2018). Replica was introduced into the model to account for any random effect. Data were also pooled over replicates, and the index of sexual isolation, I_PSI_, was calculated using the program JMating (Rolán‐Alvarez & Caballero, 2000). Positive I_PSI_ values are indicative of positive assortative mating and suggest premating isolation. Statistically significant deviations from random mating (i.e., I_PSI_ = 0) were determined by bootstrapping 10,000 in JMating.

We tested fecundity of crosses between individuals of the same population as well as between individuals of different subspecies. We followed a protocol described in Gomes and Civetta (2014). Briefly, five 5‐ to 6‐day‐old naïve males and virgin females were placed together for 48 hr in a vial containing CYMA food. Males were removed after 48 hr and females transferred to a fresh vial five days after the initial setup. Females were discarded after 5 days and progeny counted from both vials 23 days after the initial setup. Each cross was replicated at least 5 times.

### 2.3 Interrupted matings

Virgin male and female pairs of the same strain/population were aspirated into vials without anesthetization. Similarly, sterile hybrid males were paired with females of each population used to generate them. Vials containing a single male and female pair were observed continuously for a period of 3–4 hr. Mating pairs were stopped by freezing at either 2 or 6 min into copulation and stored frozen for later dissection. The frozen couples were retrieved from the freezer and allowed to briefly thaw over the course of a few minutes. All dissections were done in a drop of 1 × phosphate‐buffered saline (PBS). The frozen copulating pair was gently separated using forceps and the male was checked for an ejaculate mass on the tip of its aedeagus, in case the separation had pulled it from the female, before being discarded. The removal of the female reproductive tract was done following a protocol described by Adams and Wolfner (2007). Briefly, forceps and pins were used to separate cuticular tissue and open the abdomen. Once the uterus was visible, we gently removed it from the abdomen without disturbing the ejaculate when present. Pins were used to clean excess tissue; no coverslips were placed over samples, and images were captured using an inverted Olympus CKX41 microscope. The samples were checked for morphological shifts in the female's uterus and the presence of a darker mass that denoted an ejaculate. When present, ejaculates were removed from the female's uterus using dissecting pins and placed on a drop of NucBlue Fixed Cell DAPI stain (Thermo Fisher Scientific). A coverslip was placed over the drop containing the ejaculate and incubated in the dark for 30 min. The samples were observed for the presence of sperm under both phase contrast and UV light using a Zeiss AX10 microscope.

### 2.4 Testes’ measures of sterile and fertile males

Males from each strain as well as sterile hybrid males were collected and kept in vials containing CYAM medium with no more than 20 males per vial to avoid crowding. The males were aged to either 5 or 10 days old before being frozen to preserve them. Frozen males were allowed to briefly thaw over the course of a few minutes and dissected in a drop of 1 × PBS. Forceps were used to grip the thorax, while the other pinched the male's abdomen just above the aedeagus. Then, by simply pulling on the gripped abdomen the entire male's reproductive tract was pulled out into a drop of 1 × PBS. The testes were isolated using dissecting pins and moved onto a fresh drop of 1 × PBS. Testes’ images were captured using an inverted Olympus CKX41 microscope, and the image processing and analysis software Image J (https://imagej.nih.gov/ij/) was used to measure an area toward the basal end of the testes where mature sperm is found and which we refer to as the seminal vesicle. The area can be approximately identified by an apical pinch followed by a basal pinch or torsion. One testis from each male was measured.

### 2.5 Statistical analysis

Data from the interrupted matings were analyzed using Fisher's exact test on nominal variables (i.e., presence vs. absence). One‐way analysis of variance (ANOVA) was used to compare differences in fecundity and on area measures of testes. If significant differences were detected by ANOVA, Scheffe's post hoc test was used to determine whether the variance was statistically significant between specific samples (e.g., *Drosophila* strains). All analysis was done using SPSS software.

## 3 RESULTS

### 3.1 Positive assortative mating among populations and no evidence of noncompetitive postmating prezygotic isolation

We found significant deviation from random mating when we fit a GLMM with replicas as a random variable and analyzed the data by grouping assays using strains of different subspecies (heterotypic: wil(P) × win(S) and wil(G) × win(U); *p* < .001) or by grouping assays that used strains of the same subspecies (homotypic: win(S) × win(U) and wil(G) × wil(P); *p* < .001). Similarly, significant results were found when the analysis was conducted independently for each pair of strains, except for the homotypic cross wil(G) × wil(P) (Table 1). This departure from random mating indicates strong premating isolation between the subspecies, and positive assortative mating among populations of the same subspecies. An interesting observation is that geographically distant populations of the same subspecies (Saint Vincent and Uruguay) show evidence of assortative mating, but not geographically closer island populations (Guadeloupe and Puerto Rico) (I_PSI_ = 0.20; *p* = .145; Table 1). Altogether, premating isolation does not appear to be driven by the subspecies status of the strains assayed but rather by its population origin.

**TABLE 1:** Number of matings observed in multiple‐choice trials between homotypic and heterotypic populations

Strain A × Strain B	Mating type	A_♀_×A_♂_	A_♀_×B_♂_	B_♀_×A_♂_	B_♀_×B_♂_	I_PSI_
win(S) × win(U)	Homotypic	32	12	5	51	0.67***
wil(G) × wil(P)	Homotypic	12	18	8	27	0.20
wil(P) × win(S)	Heterotypic	50	18	18	34	0.40***
wil(G) × win(U)	Heterotypic	39	15	27	32	0.28**

Note

I_PSI_ measures deviation from random mating. P‐values were determined by bootstrapping 10,000 times (*p < .05; **p < .01; ***p < .001). win(S)= Saint Vincent; win(U)= Uruguay; wil(G)= Guadeloupe; wil(P)= Puerto Rico.

**FIGURE 1:**
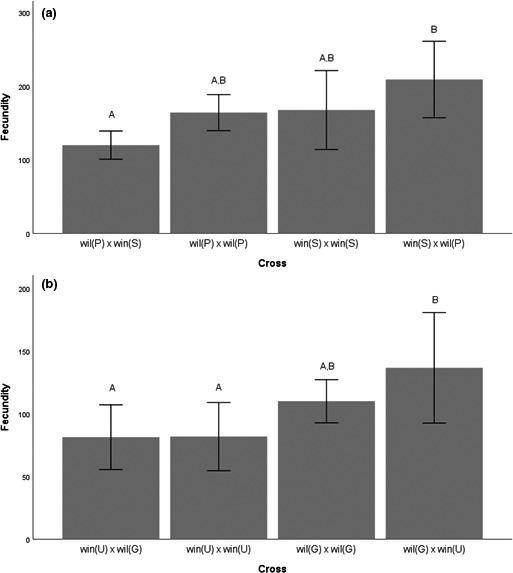
Average fecundity, and 95% confidence interval, of females mated to males of the same subspecies or a different subspecies. (a) Results are shown for crosses using Puerto Rico (*D. w. willistoni*) and Saint Vincent (*D. w. winge*) and (b) Guadeloupe (*D. w. willistoni*) and Uruguay (*D. w. winge*) flies. Shared letters above bars indicate that the groups did not differ significantly

We compared overall fecundity of females crossed to males of the same population to estimates from crosses between individuals of different subspecies. This is a noncompetitive setting, as females were not offered an opportunity to doubly mate with both males of the same population and of a different population or subspecies. We found significant differences among crosses in fecundity (*F*_7,37_ = 9.8; *p* < .001). Crosses among individuals of the same populations were nonsignificantly different from crosses between individuals of different subspecies (Figure 1).

### 3.2 Sterile hybrids and fertile males transfer seminal products that trigger morphological changes in the uterus

**FIGURE 2:**
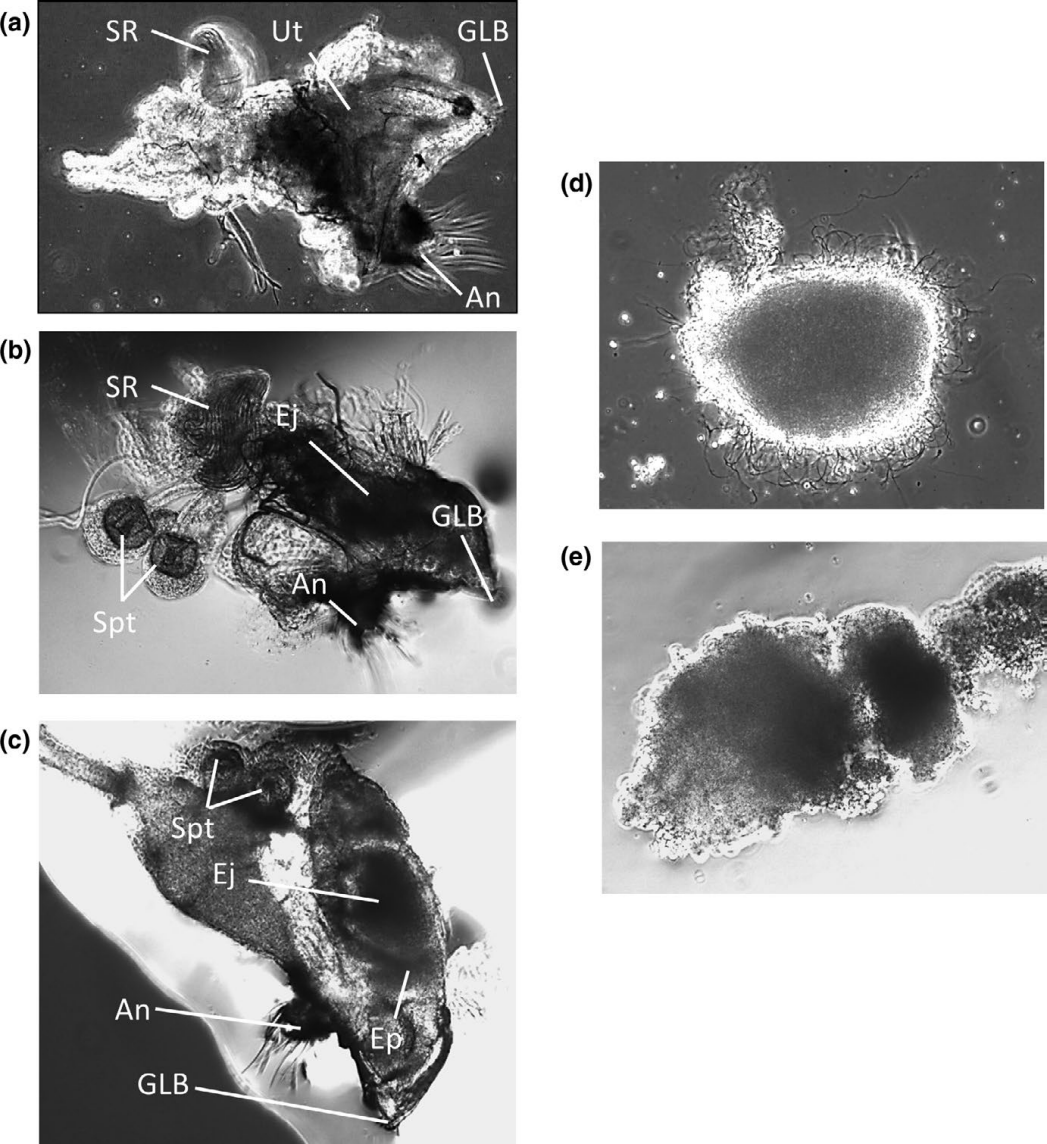
Expansion of the uterus (Ut) as a result of mating and content of the male transferred ejaculate. (a) In virgin females, the uterus (Ut) is compact between the gonopod long bristles (GLB) and the sperm storage organs (SR). (b) Two minutes into copulation, the uterus is semi‐elongated, and the ejaculate (Ej) has been transferred. (c) Six minutes into copulation, the uterus is fully elongated, and an ejaculate plug (Ep) has formed in the posterior part of the uterus. (d) Ejaculate removed from the uterus of a female mated to a fertile male. Sperm tails (black thin lines) are clearly visible. (e) Mass of seminal fluid removed from the uterus of a female mated to a sterile hybrid male is devoid of sperm. SR = seminal receptacle; Spt = spermathecae; An = anus

Interrupted copulations showed significant differences between males from parental population and sterile male hybrids in proportion of seminal fluid masses present at two minutes interruptions (parentals = 87.5%; sterile males = 33.3%; Fisher's exact test *p* = 1.3 × 10^−8^), but by six minutes, the differences were nonsignificant (parentals = 97.7%; sterile males = 86.5%; Fisher's exact test *p* = .068) (Table 2). This result shows that sterile males take longer, but effectively transfer seminal fluids. Moreover, the transfer of seminal fluids triggers similar morphological responses in the female reproductive tract of females mated to fertile or sterile males. The uterus is compacted in virgin females, with the seminal receptacle located ventrally (Figure 2a). By two minutes, the uterus experiences partial elongation acquiring a more oval shape and the seminal receptacle is displaced opposite to the gonopod (Figure 2b). At this point, the seminal fluid and possibly sperm (the ejaculate) can be sometimes detected as a darker mass inside the uterus (Figure 2b). By six minutes, the uterus is fully elongated, with an oval shape, and both the ejaculate mass and the ejaculate plug are visible (Figure 2c). In females mated to sterile males, besides taking longer for the seminal fluid mass to be transferred (Table 2), a major noticeable difference is the lack of sperm within the seminal fluid mass transferred by sterile males into the uterus (Table 2) (Figure 2d and e).

**TABLE 2:** Interrupted matings of *D. willistoni* at different times during copulation between flies from parental populations (P) and mattings involving sterile hybrids (SH)

Time	Cross	Prop SF	*p*‐value	Prop Sperm	*p*‐Value
2 min	P	0.875 (48)	<.0001*	0.98 (42)	<.0001*
	SH	0.333 (57)		0.00 (19)	
6 min	P	0.977 (43)	.068	1.00 (42)	<.0001*
	SH	0.865 (52)		0.00 (45)	

Note

Proportion of females with visible seminal fluid mass inside the bursa (Prop SF) and with sperm within the ejaculate (Prop Sperm) are shown. *p*‐Values are from Fisher's exact test comparisons between proportions in parental crosses (P) and crosses with sterile male hybrids (SH).

### 3.3 Failure to transfer sperm by sterile hybrids is caused by a testis's blockage at its basal end

**FIGURE 3:**
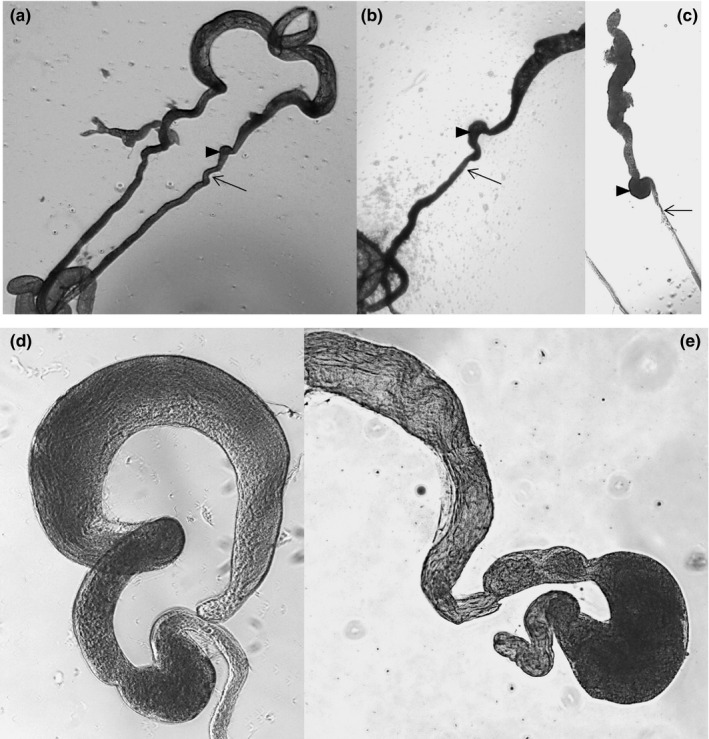
Testes of fertile and sterile hybrid males. (a) and (b) Testes of a 5‐day‐old fertile male and (c) testes of a 5‐day‐old sterile male. Arrowheads indicate the seminal vesicle, and the thin arrow, the *vas deferens*. (d and e) Testes of a 5‐ and 10‐day‐old sterile male showing enlargement of the seminal vesicle

Visual inspection of the testes identified a region toward its basal end that looked enlarged and more oval in sterile hybrid males than fertile parentals (Figure 3). Because this structure contains individualized sperm, we refer to it as the seminal vesicle. In 5‐day‐old fertile males, a pinch in the testes often defines the apical end of what we identified as the seminal vesicle (i.e., mature sperm storage) with the basal end almost continuously extending into a tubular structure that we refer to as the *vas deferens* (Figure 3a). The basal end of the vesicle can be harder to identify in fertile males, in some a subtle torsion or pinch, and the presence of a darker mass of sperm can be used as a landmark (Figure 3a), while in others the basal end is more distinguishable (Figure 3b). In both cases, the vesicle continues into a thicker tubular structure (Figure 3a and b) compared with sterile males (Figure 3c). In sterile hybrids, the seminal vesicle is more clearly distinguishable by an apical and basal pinch, and it is more oval and enlarged (Figure 3c) compared with the more elongated form found in the fertile testes. We used the landmarks described above to delimit this structure and to provide approximate measurements of its area in fertile and sterile males of different ages. Analysis of variance showed significant differences in the size of the seminal vesicle between fertile parental males and sterile hybrids at five (*F*_1,59_ = 148.6; *p* < .001) and ten days of age (*F*_1,59_ = 379.5; *p* < .001) (Table 3). When the analysis is partitioned by population types (Uruguay, Guadeloupe, Saint Vincent, and Puerto Rico), Scheffe's post hoc tests showed that parental populations are significantly smaller than sterile male hybrids at all ages and not significantly different from each other. In sterile hybrids, the seminal vesicle is approximately two times larger than in fertile males and grows as males ages (Table 3; Figure 3d and e).

**TABLE 3:** Measurement of the area of the male seminal vesicle (in mm^2^ × 1,000) in 5‐ and 10‐day‐old sterile male hybrids (SH) and fertile parental (FP) males. Sample size is shown in parenthesis

Male	5 days	10 days
FP wil(G)	14.1 (10)	21.2 (10)
FP win(U)	18.2 (10)	22.8 (11)
FP wil(P)	18.8 (9)	30.9 (10)
FP win(S)	17.6 (10)	23.0 (10)
Avg. FP	17.2 (39)	24.5 (41)
SH wil(G) × win(U)	31.9 (12)	54.5 (9)
SH wil(P) × win(S)	31.3 (10)	61.3 (11)
Avg. SH	33.3 (22)	57.9 (20)
Ratio of SH/FP	1.9	2.4

**FIGURE 4:**
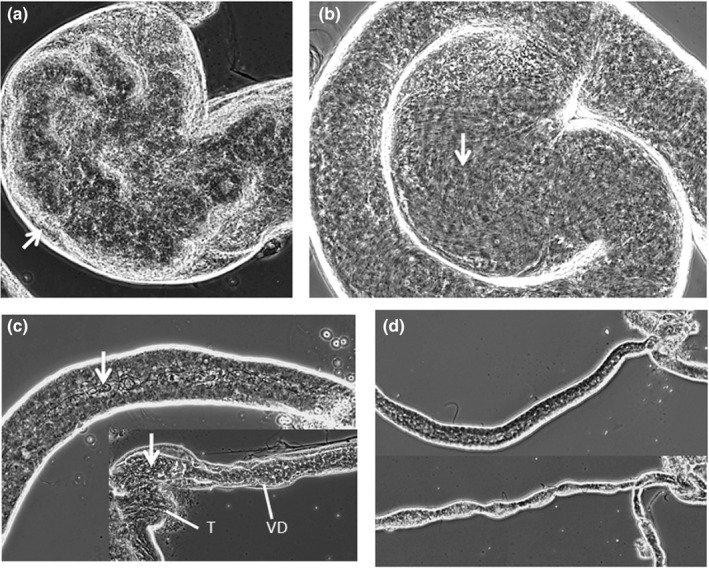
Seminal vesicle and *vas deferens* of fertile and sterile hybrid males. (a) Seminal vesicle of a sterile hybrid male showing vacuolar formations and sperm (arrow) displaced to the edge. (b) Seminal vesicle of a fertile male with arrow pointing at sperm. (c) *Vas deferens* (VD) of a fertile male (above) with sperm (arrow) present in its lumen versus sperm (arrow) within the testes (T) of a sterile hybrid not entering the VD. (d) VD of a fertile male (above) versus sterile hybrid (bellow) showing an irregular shape and lack of lumen

Closer examination of the seminal vesicle and *vas deferens* allowed us to find some interesting structural abnormalities in the sterile hybrids that might at least partially explain how the enlargement of the seminal vesicle might affect the transfer of sperm: (a) The seminal vesicle is more vascularized in sterile males, with sperm present but displaced to the edges (Figure 4a), (b) sperm is not found in the *vas deferens* of sterile hybrids (passing a pinch at the base of the seminal vesicle, which is enlarged in sterile males), suggesting that the enlarged and vascularized structure impedes proper movement of sperm into the *vas deferens* (Figure 4b), and (c) some level of atrophy of the *vas deferens* in sterile hybrid males is apparent by the observation of its irregular shape and the lack of lumen suggesting collapsing and obstruction of the duct (Figure 4c).

## 4 DISCUSSION

We found evidence of deviation from random mating for both homotypic and heterotypic crosses and premating isolation among populations of *D. willistoni* that is not determined by the subspecies status. Given the costly consequences of producing sterile male hybrids in crosses among populations of different subspecies, we expected premating barriers might exist due to selection against maladaptive hybridization in heterotypic crosses (Coyne & Orr, 2004). However, the occurrence of premating isolation in crosses involving populations of the same subspecies is rather unexpected. While it is possible that premating reproductive barriers to gene flow are important among populations of *D. willistoni* regardless of their subspecies status, a caveat is our small sample size and the use of laboratory strains that do not allow us to determine to what extent the partial but significant levels of isolation have been a consequence of laboratory conditions. While we consider it unlikely, if the levels of premating isolation we detected among populations arose or became stronger in laboratory stocks then clearly this form of isolation is not an important contributor to reproductive isolation between subspecies. If positive assortative mating among males and females of the same populations truly reflects a condition found in natural populations, then premating isolation is clearly important, but it is not a fixed condition. Contrary to premating isolation, postmating postzygotic isolation (i.e., unidirectional male hybrid sterility) is a fixed condition between subspecies (i.e., *D. w. willinstoni* and *D. w. winge*) that is unlikely to have been created in laboratory settings for two reasons: (a) The isolation mechanism is fixed in a pattern that is geographical rather than random (north vs south; Mardiros et al., 2016), and (b) the same observation of hybrid male sterility among populations of north versus south origin was made over 40 years ago by H. Winge before the laboratory stocks were established (Cordeiro & Winge, 1995; Dobzhansky, 1975).

An interesting observation regarding the levels of premating isolation detected between populations is the significant isolation between geographically distant populations (i.e., Saint Vincent and Uruguay) but not between geographically closer populations of the same subspecies (i.e., Guadeloupe and Puerto Rico). Isolation by distance rather than between geographically closer populations suggests that allopatry might have facilitated the evolution of positive assortative mating among populations, rather than premating barriers being reinforced upon possible secondary contacts among more geographically closer populations. This pattern of increased isolation by distance is preliminary given the small sample sizes but reminiscent of observations made in other populations of *Drosophila* (Garlovski & Snook, 2018; Jennings et al., 2014) and, in our case, suggests that postmating isolation might be particularly important as a barrier during early divergence of these two subspecies of *D. willistoni*.

We have shown that sterile males manage to trigger changes in the morphology of the female's uterus that is not different from the changes induced by fertile males. While the transfer of the seminal fluids seems to be slower in sterile males, their ability to cause the same morphological changes as fertile males suggests no major differences among subspecies in seminal fluid composition. We know from some studies in *D. melanogaster* that interactions between components of the male seminal fluid and the female reproductive tract trigger female responses to mating and are needed for efficient fertilization (Avila & Wolfner, 2017; Chen et al., 2019; Rezával et al., 2012). Often, male seminal fluid proteins are rapidly evolving among species (reviewed in Swanson & Vacquier, 2002) and can potentially contribute to postmating barriers between species (Castillo & Moyle, 2014). Our findings suggest that components of the seminal fluids responsible for morphological changes after mating are conserved, and our results from fecundity assays show no evidence for noncompetitive postmating prezygotic isolation between populations of different subspecies. However, it is possible that proteins involved in competitive processes might have substantially diverged between these subspecies. Therefore, we cannot rule out the possibility that competitive postmating prezygotic isolation (e.g., conspecific sperm precedence) might exist between these two subspecies.

Problems in sperm transfer have been reported before, for example, *Drosophila simulans* females mate for a shorter period of time with *Drosophila sechellia* males than with conspecific males and very few sperm are transferred (Price, Kim, Gronlund, & Coyne, 2001). However, complete failure to transfer sperm as a form of hybrid male sterility imposing postzygotic isolation between recently diverged subspecies has not been previously reported among species of *Drosophila*. It has been previously shown that hybrid sterile males between the *D. w. willistoni* and *D. w. winge* produce normal and fully motile sperm (Gomes & Civetta, 2014) and we show here that hybrid male sterility results from a blockage impeding movement of sperm from the seminal vesicle into the *vas deferens* and mixing with other components of the ejaculate. The faster male hypothesis predicts that given sexual selection among males, there should be faster divergence of genes contributing to sperm development (Wu & Davis, 1993). Thus, hybrid male sterility at early stages of speciation caused by blockage originating during tissue development rather than divergence of the germ line is unexpected.

The type of failure to transfer sperm due to a blockage (azoospermia) we report for flies is reminiscent of cases of sterility in humans (Jarvi et al., 2010) and might be a consequence of abnormalities during the male reproductive tract developmental process. The development of the male reproductive tract during pupation is split into two parts, the genital disk which leads to the formation of most of the internal organs and the external genitalia, and the gonads which develop into the testes. Both the *vas deferens* and the seminal vesicles arise from the genital disk, and the proper development of the testes depends on fusion with the seminal vesicles (Rothenbusch‐Fender et al., 2017). Given the overall normal male reproductive tract morphology of the sterile hybrids, we can conclude that the fusion occurs successfully. Based upon reports on the reproductive tract formation of *Drosophila melanogaster*, the seminal vesicles form prior to the *vas deferens* (Kuckwa, Fritzen, Buttgereit, Rothenbusch‐Fender, & Renkawitz‐Pohl, 2016; Rothenbusch‐Fender et al., 2017). It is feasible that the origin of the enlargement in sterile hybrids be a consequence of subtle abnormalities at or around the time when the seminal vesicles are formed. Given that smooth musculature grows over the testes, it is possible that the musculature layer of the *vas deferens* grows, but the interior lumen is hindered by a defect in the seminal vesicle.

Here, we have shown that noncompetitive postmating prezygotic isolation is not a barrier to hybridization between *D. w. willistoni* and *D. w. winge*, but incomplete premating isolation is detectable among populations regardless of subspecies status. We have characterized a unique form of hybrid male sterility that involves an impediment of the male's ability to transfer sperm. Detail characterization of the “speciation phenotype” is crucial in guiding future attempts to understand its genetic basis.

## CONFLICT OF INTEREST

The authors declare no conflict of interest.

## AUTHOR CONTRIBUTIONS

**Hunter Davis:** Conceptualization (supporting); Formal analysis (supporting); Investigation (lead); Methodology (lead); Writing‐original draft (lead). **Nicholas Sosulski:** Conceptualization (supporting); Formal analysis (supporting); Investigation (supporting); Methodology (supporting); Writing‐original draft (supporting). **Alberto Civetta:** Conceptualization (lead); Formal analysis (lead); Funding acquisition (lead); Investigation (supporting); Methodology (supporting); Supervision (lead); Writing‐original draft (supporting); Writing‐review & editing (lead).

## Data Availability

Data supporting this publication are available at the Dryad Digital Repository (https://doi.org/10.5061/dryad.wwpzgmsgd).
